# Novel Combination Therapies for the Treatment of Bladder Cancer

**DOI:** 10.3389/fonc.2020.539527

**Published:** 2021-01-27

**Authors:** Mei Peng, Di Xiao, Yizhi Bu, Jiahui Long, Xue Yang, Shuhe Lv, Xiaoping Yang

**Affiliations:** ^1^ Department of Pharmacy, Xiangya Hospital, Central South University, Changsha, China; ^2^ Key Laboratory of Study and Discovery of Small Targeted Molecules of Hunan Province, Department of Pharmacy, School of Medicine, Hunan Normal University, Changsha, China

**Keywords:** combination therapies, immunotherapy, targeted therapy, chemotherapy, bladder cancer

## Abstract

Bladder cancer is the ninth most frequently diagnosed cancer world-wide and ranks 13th in cancer-related deaths. Two tremendous breakthroughs in bladder cancer therapy over the last decades are the approval of immune checkpoint inhibitors(ICIs)and the fibroblast growth factor receptor tyrosine kinase inhibitor (FGFR-TKI) erdafitinib for treating this deadly disease. Despite the beneficial effects of these approaches, the low response rate and the potential resistance of the cancer are major concerns. Hence, novel combination therapies to overcome these limitations have been investigated. In this context, combining immunotherapy with targeted drugs is an appealing therapeutic option to improve response and reduce the emergence of resistance in the management of bladder cancer. In this review, the rationale of using different therapeutic combinations is discussed according to the mechanistic differences, emphasizing the efficacy and safety based on evidence collected from preclinical and clinical studies. Finally, we highlight the limitations of these combinations and provide suggestions for further efforts in this challenging field.

## Introduction

Bladder cancer is one of the most prevalent cancers worldwide, with around 430,000 new diagnoses and 150,000 deaths each year (1 **)**. Approximately 75% of newly diagnosed patients have non-muscle invasive bladder cancer (NMIBC) with standard treatment of intravesical chemotherapeutic drugs or immune inhibitor after tumor resection **(** 2 **)**. However, around 40-50% of patients will experience recurrence within five years of diagnosis with up to 80% in the highest-risk groups (2 **)**. The remaining 25% of patients have muscle invasive bladder cancer (MIBC) or metastatic disease and the gold standard therapeutic method is radical cystectomy followed with systemic chemotherapy. However, prognosis in this population of bladder cancer patients is poor and the 5-year overall survival (OS) rate is 15% (3, 4).

The past decade has witnessed the rapid development of combination therapy for improved therapeutic outcomes in bladder cancer. Combination therapy has been a successful strategy to enhance efficacy, increase response, reverse resistance and reduce toxicity as well as address tumor heterogeneity upon using different drugs of different dynamics and molecular targets (5, 6). Previously, the common combination regimens are drugs composed of different chemotherapeutic anticancer drugs or combining chemotherapy with radiotherapy. With comprehensive analysis of bladder cancer cases, molecular characterization is figured out and this provides rationales for novel therapies. NMIBC is primarily presented with FGFR3 alterations while MIBC has a more diverse mutation spectrum (7, 8 **)**. High mutational burden in bladder cancer provides implications for the use of targeted and immune checkpoint inhibitors (ICIs). Recently, immunotherapy has become a hot topic since the approval of programmed death-1 (PD-1)/programmed death-1 ligand 1 (PD-L1), cytotoxic T lymphocyte-associated antigen-4 (CTLA-4) ICIs by the U.S. Food and Drug Administration (FDA)with satisfying efficacy in advanced cancers **(** 9, 10 **).** However, low response rate, emergence of drug resistance, and tolerability concerns appeared quickly. The tumor microenvironment significantly influences therapeutic response and efficacy. Thus, combination therapy *via* regulation of immune microenvironment for the purpose of sensitizing drug activity and decreasing doses has been under investigation (11 **)**. Another breakthrough is the introduction of erdafitinib, an oral pan-FGFR-targeted tyrosine kinase inhibitor approved by the US FDA in 2019 for treatment of metastatic urothelial carcinoma (UC) patients with susceptible FGFR3 or FGFR2 alterations (12).

In this mini-review, multiple combination regimens including chemotherapy, radiotherapy, targeted therapy, and immunotherapy for treating bladder cancer in preclinical or clinical settings are discussed. This review will provide a comprehensive summary for readers to understand the present and future potential combination therapies in bladder cancer.

## Immunotherapy

Immune checkpoints refer to inhibitory pathways built into the immune system which are vital to limit collateral tissue damage (that is, the prevention of autoimmunity) under the circumstance of physiological immune responses (13 **)**. Immune checkpoints are initiated by ligand-receptor interactions. For example, normal cells harbor PD-L1 bind to PD-1 receptors on T-cells to suppress excessive immune response (14 **)**. In addition, the activation of the receptor CTLA-4 located in T cells inhibits the initiation of the immune response by T cells, resulting in the reduction of activated T cells and preventing the formation of memory T cells (15 **)**. However, Tumor cells can up-regulate PD-L1 or activate CTLA-4 and this ligand-receptor binding causes inactivation of T cells and tumors escaping the immune response (16 **)**. Therefore, the FDA approved ICIs that block the interaction between CTLA-4 and its ligand or block the interaction between PD-1 and PD-L1, thereby restoring cytotoxic T cell immune response in recognizing and destroying cancer cells thus preventing growth of tumors (9, 10 **)**. Immunotherapy is approved as a second-line treatment for metastatic urothelial cancer (17 **)**. Their use as a first-line agent is only limited to patients who are ineligible for cisplatin-based treatments (17 **)**. There is a biological and clinical rationale for using immunotherapy in NMIBC patients. First, the historic use of bacillus Calmette−Guerin(BCG)in NMIBC attests to the effectiveness of immunotherapy for these patients and supports evaluation of other immunotherapy strategies to overcome resistance to BCG. Second, it is well known that genomic and epigenomic alterations drive the pathogenesis of bladder cancer (18 **)**, with many alterations thought to provide neoantigens that may elicit potent antitumor immune responses (8, 18). High-grade NMIBC harbors many of the same genomic alterations as muscle invasive and metastatic bladder cancer (8 **)**. Tumors with a higher mutational load produce many neoantigens that are recognized as foreign by the immune system, thereby triggering a T-cell mediated antitumor immune response (19 **)**. High mutational burden has also been associated with increased efficacy of ICIs (20, 21). From a preclinical perspective, evidence from bladder cancer models in immunocompetent mice supports the use of ICIs alone or in combination with other treatment modalities in bladder cancer (22 **)**. From a clinical context, the approval of five inhibitors of the PD-1/PD-L1 axis (atezolizumab, pembrolizumab, nivolumab, durvalumab, and avelumab) for the treatment of advanced or metastatic UC provides a compelling and logical rationale for testing checkpoint blockades in the earlier stage, BCG-unresponsive NMIBC. Although immunotherapy is better tolerated than chemotherapy, autoimmune side effects are be particularly concerning. Simultaneously, based on results from clinical trials, the overall response rate of immunotherapy is ranging from 17% to 23% and indicating that immunotherapy is only effective for a minority of patients. Thus, there is an urgent need to find new therapeutic approaches to improve response rates. Combinations of immunotherapy with conventional agents are being investigated in several preclinical and clinical studies in urothelial cancer. **Table 1** summarizes ongoing clinical trials for ICIs and other novel combination therapies for the management of bladder and urothelial cancers.

**Table 1:** Ongoing clinical trials of novel combination therapies in bladder cancer and urothelial cancer.

**Study number**	**Eligibility**	**Phase**	**Intervention**
NCT01928394	Advanced or metastatic bladder cancer	Phase I/II	Nivolumab and Ipilimumab
NCT03084471	Advanced bladder cancer	Phase III	Durvalumab and Tremelimumab
NCT03219775	Metastatic or advanced transitional cell carcinoma	Phase II	Nivolumab and Ipilimumab
NCT03036098	Unresectable or metastatic UC	Phase III	Nivolumab, Ipilimumab, Gemcitabine/Cisplatin/Carboplatin
NCT02516241	Unresectable stage IV UC	Phase III	Durvalumab and Tremelimumab
NCT04223856	Advanced or metastatic UC	Phase III	EV and Pembrolizumab
NCT03519256	BCG unresponsive high-risk NMIBC	Phase II	Nivolumab, BMS-986205 and BCG
NCT02560636	Advanced bladder cancer	Phase I	Pembrolizumab and radiotherapy
NCT02643303	Bladder cancer	Phase I/II	Tremelimumab, Durvalumab and polyICLC
NCT03473743	Metastatic or advanced UC	Phase Ib-II	Erdafitinib, Cetrelimab and Platinum
NCT03473756	UC	Phase Ib/II	Rogaratinib and Atezolizumba
NCT04172675	High-risk NMIBC	Phase II	Erdafitinib, Gemcitabine/Mitomycin C
NCT03745911	Metastatic UC	Phase II	Paclitaxel and TAK-228
NCT02546661	MIBC	Phase I	Durvalumab, Olaparib, AZD1775 and Vistusertib
NCT03022825	BCG unresponsive high grade NMIBC	Phase II	ALT-803 and BCG

BCG, bacillus Calmette-guerin vaccine; EV, enfortumab vedotin; MIBC, muscle-invasive bladder cancer; NMIBC, non-muscle-invasive bladder cancer; polyICLC, polyinosinic-polycytidylic acid-poly-l-lysine carboxymethylcellulose; UC, urothelial carcinoma.

### Combination of PD-1 With CTLA-4 Inhibitors

It is well established that tumors use PD-1 and CTLA-4 pathways to silence the immune system (16 **)**. The CTLA-4 antibody promotes the entry of anti-cancer immune cells into the surrounding tumor tissue and eliminates the immunosuppressive cells that promote cancer growth (23 **)**. At the same time, the role of PD-1 antibody is to activate these immune cells to prevent tumor cell immune escape (16 **)**. Recent data have shown that combination therapy with an anti-PD-1 and anti-CTLA-4 antibodies demonstrated significant preclinical and clinical responses in bladder cancer (24). Duraiswamy et al. provided evidence that reversal of T-cell dysfunction could be achieved by simultaneously targeting effector T cells and regulatory T cells (Tregs). The study showed that co-expression of both PD-1 and CTLA-4 was associated with marked dysfunction of antigen-specific T cells so blockade of PD-1 and CTLA-4 pathways reversed T-cell dysfunction. It proved that adoptive transfer tumor-infiltrating lymphocytes (TIL) that had been pretreated *in vitro* with anti-PD-1 and anti-CTLA-4 antibodies eliminated tumors *in vivo* (25). Furthermore, immunohistochemistry staining for CD3+ T cells in the MC38 tumor model revealed the highest CD3+ T-cell tumor infiltration in the anti-CTLA-4/PD-1 monoclonal antibodies combination setting (26). Higher tumor infiltration likely accounts for CTLA-4/PD synergy. Shi et al. elucidated the underlying tumor rejection mechanisms for the combination therapy of PD-1 with CTLA-4 inhibitors by performing a detailed analysis of human bladder tumor samples together with murine MB49 bladder tumor model (27). The results showed that combination therapy improved tumor rejection by promoting T-cell infiltration into tumors, encouraging the proliferation and polyfunctionality of TILs, and endogenous memory T cells expansion. The interactions among these immune cells are mediated by the interdependent loop between interleukin-7 (IL-7) and interferon gamma (IFN- γ) signaling (27). These provided direct evidence that additional blockade of PD-1 hindered tumor from breaking away from an anti-CTLA-4 inhibitor monotherapy and additional blockade of PD-1 handicapped tumor from getting rid of a-CTLA-4 monotherapy *via* protecting immunity by both T-cell-dependent, and natural killer (NK)/natural killer T (NKT) cell-independent fashions (27). In clinical trials, current PD and CTLA-4 combinations are paired as durvalumab/tremilimumab and nivolumab/ipilimumab. CheckMate-032 assessed nivolumab monotherapy and two combinations of nivolumab and ipilimumab in participants with platinum-refractory advanced bladder cancer. The dosage was variant in the combination groups, with nivolumab 1 mg/kg + ipilimumab 3 mg/kg (n = 26) in one cohort and nivolumab 3 mg/kg + ipilimumab 1 mg/kg (n = 104) in the other. From preliminary data, OS and objective response rate (ORR) were stronger in the cohort receiving a greater ipilimumab dose (10.2 months and 39%) compared to nivolumab monotherapy or the other combination (7.3 months and 26%) (28 **)** (NCT01928394). Optimal sequencing is being tested in TITAN-TCC, in which subjects begin with nivolumab monotherapy induction and, should no response occur, receive boost cycles of nivolumab/ipilimumab (NCT03219775). Potential utility in the first line is being tested in CheckMate-901, previously discussed for its gemcitabine + cisplatin (GC)+ nivolumab arm, is also testing nivolumab/ipilimumab. This combination will be assessed against standard of care (SoC) chemotherapy, and the study aims for an enrollment of 897 (NCT03036098). Multiple umbrella trials are investigating durvalumab/tremelimumab together in advanced cancers. STRONG (NCT03084471) compares a fixed dose regimen of durvalumab 1500 mg + tremelimumab 75 mg to durvalumab 1500 mg monotherapy in advanced cancers including bladder cancer. Subjects will have progressed on prior chemotherapy. Durvalumab/tremelimumab is being compared to SOC chemotherapy in a phase III trial dubbed DANUBE (NCT02516241). Stage IV bladder cancer patients formed the study’s population (est. n = 1200) and were randomized 1:1:1 to durvalumab monotherapy, durvalumab with tremelimumab, or chemotherapy **(** 29 **)**. This combination was assessed in a phase I/II study of advanced cancers including bladder cancer with the addition of intra-tumoral polyinosinic-polycytidylic acid-poly-l-lysine carboxymethylcellulose, which is a toll-like receptor 3 agonist and a modulator of the tumor microenvironment (NCT02643303).

### Combination of Radiation Therapy With Immunotherapy

Radiation therapy (RT) has evolved over the past several decades as a powerful way to treat cancer (30 **)**. However, it has some limitations as it alone cannot generate a systemic effect. Integration of RT with the immunotherapies has been a subject of intense research recently. The rationale behind the combination was initially derived from abscopal effect observations. It is a phenomenon whereby radiation at one site leads to the regression of metastatic cancer at a distant site that has not been exposed to any radiation (31 **)**. Advances in immunology have progressed our understanding of the phenomena, and while the mechanism is still not entirely elaborated, the explanation for combining immunotherapy and radiation to increase the frequency of the abscopal effect is irradiation activated cytotoxic T cells to target tumor-associated antigens (TAAs) within human bodies, thereby overcoming the immunosuppressive tumor microenvironment. Furthermore, radiotherapy might increase the response rate of combination by stimulating the accumulation and activation of CD8 + T cells (32 **)** to create a more permissive tumor microenvironment.

**Figure 1:**
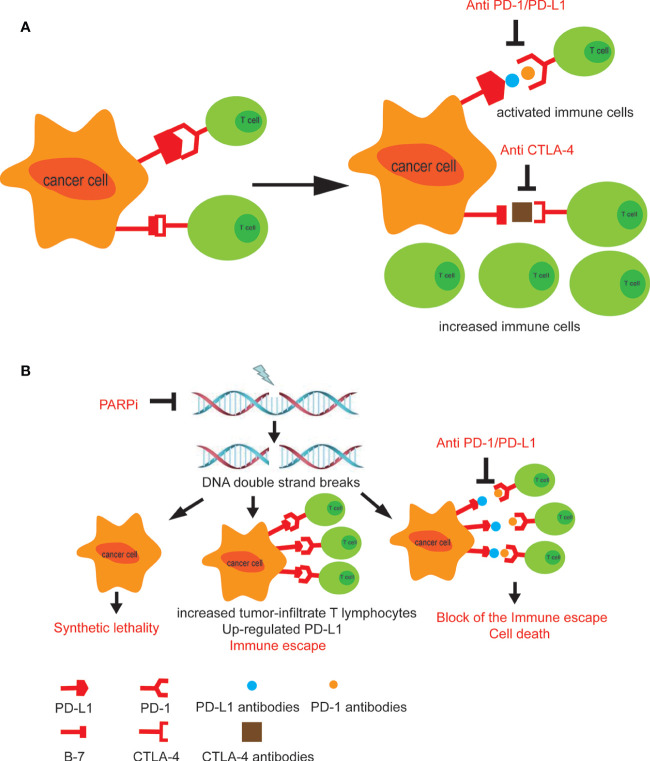
**(A)** The combined use of CTLA-4 and PD-1 inhibitors promotes anti-cancer immune cells to enter the surrounding of the tumor tissue and activates immune response. **(B)** The treatment of PARP inhibitors leads to PD-L1 upregulation in tumor cells. Combining PARP inhibitors with immunosuppressants blocks tumor immune escape (39).

Preclinical and clinical trials showed that the combination of the immunotherapy and RT had the potential to provide a synergistic effect in treating cancer, including NMIBC (33 **)**. Interestingly, T-cell activity was important for radiation efficacy in tumor control. Wu et al. found that radiation transiently increased PD-L1 expression, and PD-1 or PD-L1 blockade not only led to tumor control, but also enhanced the efficacy of RT, and the combination had increased efficacy compared with either modality alone (34 **)**. In addition to improving local control of treated tumors, several recent cases of the abscopal effect with RT published in the literature were in the setting of ICI therapy, suggesting that the combination of ICIs and RT may be the scenario where the abscopal effect may occur with a higher frequency (35, 36 **)**. Finally, the combination of immunomodulating agents and RT may result in protective immunologic memory, preventing subsequent recurrences of disease. However, there are many unanswered questions regarding the practical and logistic combination of RT and immunotherapy. For example, the optimal consequence of immunotherapy and RT, the optimal immunotherapy dose, and the duration of radiotherapy need to be clarified. Additionally, details regarding the RT, such as the optimal dose/fractionation, target volume, and site to irradiate are not known (37 **)**. Since an inappropriate combination can increase the patient’s therapeutic toxicity, the PLUMMB trial (Pembrolizumab in Muscle-invasive/Metastatic Bladder cancer) (NCT02560636) is in a phase I study to test the tolerability of a combination of weekly RT with pembrolizumab in patients with metastatic or locally advanced urothelial cancer of the bladder. In the first dose-cohort, patients received pembrolizumab 100 mg 3-weekly, starting 2 weeks before commencing weekly adaptive bladder RT to a dose of 36 Gy in 6 fractions. The first dose-cohort was stopped after 5 patients, having met the predefined definition of dose-limiting toxicity. Three patients experienced grade 3 urinary toxicities, 2 of which were attributable to therapy. One patient experienced a grade 4 rectal perforation. In view of these findings, the trial had been paused and the protocol would be amended to reduce RT dose per fraction (38 **)**. As a result, clinical trials are underway on the optimal combination of radiation and immunotherapy to treat various cancers, including bladder cancer (37 **)**. In conclusion, the combination of radiotherapy and immunotherapy has a great prospect (**Figure 1A**).

### Combination of IDO1 With Immunotherapy

Indoleamine 2,3-dioxygenase 1(IDO1) enzyme is involved in the catabolism of the essential amino acid tryptophan and plays an important role in immune evasion and tumor growth through production of kynurenine. The IDO1 enzyme is activated in many human cancers including NMIBC (40, 41 **)**. Recent data indicate that IDO1 gene expression characterizes a poorly differentiated, more aggressive NMIBC, with IDO1 gene expression in tumor tissues directly correlating with tumor size (R (correlation coefficient) =0.24, p=0.04) and stage (R=0.25, p=0.03) **(** 41 **)**. Moreover, there was a trend toward longer OS in patients with tumors that did not express IDO1. IDO inhibitors such as BMS-986205, epacadostat, indoximod, navoximod, and HTl-1090 are in various stages of clinical development in several cancers. There is evidence supporting an interrelationship between the PD-1/PD-L1 and IDO1 axes, with IDO functional activity linked with increased PD-L1 positive cytotoxic T-cells and increased CTLA4 expression by regulatory T cells (42). Therefore, it has been proposed that parallel inhibition of these pathways may lead to a more effective activation of T cell mediated antitumor immune response.

Indeed, in an advanced bladder cancer cohort (n=29) of an ongoing multi-arm, phase I/IIa dose-escalation and expansion study (CA017-003), treatment with oral BMS-986205 (100 or 200 mg once daily) in combination with nivolumab (2 schedules) resulted in an ORR of 34% and disease control rate of 48%. The ORR was 38% in patients with no prior immunotherapy (n=26), 47% in patients with tumor PD-L1 1% (n=15), and 27% in those with tumor PD-L1 <1% (n=11). The authors reported that the combination of BMS-986205 plus nivolumab was well tolerated (43 **)**. Preliminary phase I/II results of the ECHO-202/KEYNOTE-037 trial also demonstrated that oral epacadostat plus pembrolizumab was well tolerated and yielded an ORR of 35% in patients with advanced UC (43 **)**. Preliminary antitumor signals in the advanced UC cohort of the CA017-003 study and the ECHO-202/KEYNOTE-037 study are suggestive of potential activity in bladder cancer (44 **)**. Based on these data, the aforementioned CheckMate 9UT trial has been designed to investigate four different treatment regimens (nivolumab alone, nivolumab plus BCG, nivolumab plus BMS-986205, or nivolumab plus BMS-986205 and BCG) in BCG-unresponsive, high-risk NMIBC (NCT03519256)

### Combination of PARP Inhibitors With Immunotherapy

Poly ADP-ribose polymerase (PARP) inhibitors (PARPi), such as Olaparib, amplifies the DNA damage, augments the mutational burden and promotes the immune priming of the tumor by increasing the neoantigen exposure and increasing tumor-infiltrate T lymphocytes (45 **)**. Studies also reported that defects in DNA damage repair (DDR) genes could be potential predictive biomarkers of clinical response to ICIs in metastatic urothelial bladder cancer (46, 47 **)**. However, the use of PARPi can also lead to upregulation of PD-L1 in tumor cells, leading to tumor immune escape. Therefore, the combination of PARPi and immunosuppressants will benefit patients including those with bladder cancer. Interestingly, a more recent study, in 60 patients with advanced UC, had indicated that defects in DDR pathways may enrich for antitumor responses to anti–PD-1/L1 (48 **)**. In this study, patients with a deleterious alteration in at least one of 34 DDR genes showed a response rate of 80% versus only 18.8% in patients lacking these alterations. Thus, the combination of PARPi and the anti-PD/PD-L1 targeting may represent a promising strategy for bladder cancer treatment **(** 39 **)** (NCT02546661). Ross et al. summarized available data and found that combinations of PARPi and anti–PD-1/L1 agents were well tolerated and demonstrated antitumor activity in a range of tumor types (49 **)**. An open-label randomized multidrug biomarker-directed phase Ib study, the BISCAY trial, was designed to evaluate the effects of the treatments with the PARPi Olaparib as a single agent therapy, or in association with the ICI durvalumab (anti-PD-L1 antibody), for treatment of urothelial bladder cancer patients who had progressed from prior treatment and also presented defects in DNA-repair genes (NCT02546661) (**Figure 1B**).

Epigenetics is defined as a heritable modification to DNA without alteration in the nucleotide sequence, resulting in altered gene transcription and chromatin structure. Epigenetic modifications include DNA methylation and post-translational histone modifications involving methylation or acetylation are common in bladder tumors. Growing evidence showed that epigenetic drugs, such as DNA methyltransferase inhibitors can upregulate immune signaling through demethylation of endogenous retroviruses and cancer testis antigens. It provides a strong rationale for the combination of epigenetic drugs with ICIs (50, 51 **)**. Interestingly, RRx-001, not only a new DNA damage inducer, but also an epigenetic and immunomodulatory drug, has been recently investigated as single chemotherapeutic agent to re-sensitize tumor to prior therapy (52–54 **)**. The low toxicity profile of RRx-001 differentiates this agent from conventional anticancer drugs, such as chemotherapeutics, and epigenetic agents (54, 55 **)**. Indeed, RRx-001 is able to trigger DNA damage response in urothelial bladder cancer cells, reducing the DNA methyltransferase1(DNMT1) levels and increasing the transcriptional levels of the interferon type III and the interferon stimulated genes (56 **).** Thus, it enhances the sensitivity to ICIs. Criscuolo D et al. investigated the effects of combining three classes of drugs together with epigenetic agents and immune-checkpoint inhibitors in bladder cancer for the purpose of reducing toxicity and doses of monotherapy alone (39 **)**.

### Combination of Antibody-Drug Conjugates (ADSs) With Immunotherapy

The response of immunotherapy is a big concern in clinic. The combination of ADCs with immunotherapy attempts to increase patients’ overall response rate. ADCs are monoclonal antibodies conjugated to cytotoxic agents through a chemical linker, which can achieve selective targeting of cancer cells (57 **)**. In December 2019, the United States Food and Drug Administration (FDA) approved the first ADCs, enfortumab vedotin (EV), for the treatment of platinum-refractory and immune checkpoint blockade-refractory locally advanced or metastatic UC. A phase I study of EV in 112 patients with immunotherapy and platinum refractory metastatic UC treated at the 1.25mg/kg dose level indicate a 43% confirmed ORR, including five complete responses (50 **)**.

A phase 1b study (58 **)** investigating combination of EV (1.25 mg/kg) plus pembrolizumab (200mg) for cisplatin-ineligible patients with metastatic UC. The preliminary data showed that patients tolerated it well and achieved a response rate of 73.3% (59 **)**. Based on the efficacy observed in the trial, a randomized phase III study (NCT04223856) of EV and pembrolizumab with or without platinum-based chemotherapy for the first-line treatment of locally advanced or metastatic urothelial cancer was initiated (60 **)**.

## Targeted Therapy

Targeted therapy is a revolutionary treatment which can prevent the growth, progression, and metastasis of cancer by interfering with specific molecules. This therapy has achieved satisfactory results in the treatment of various cancers, such as breast cancer and colon cancer (61). However, the contribution of targeted drugs in UC is very limited due to the lack of efficacy or treatment-related toxicity.

**Figure 2:**
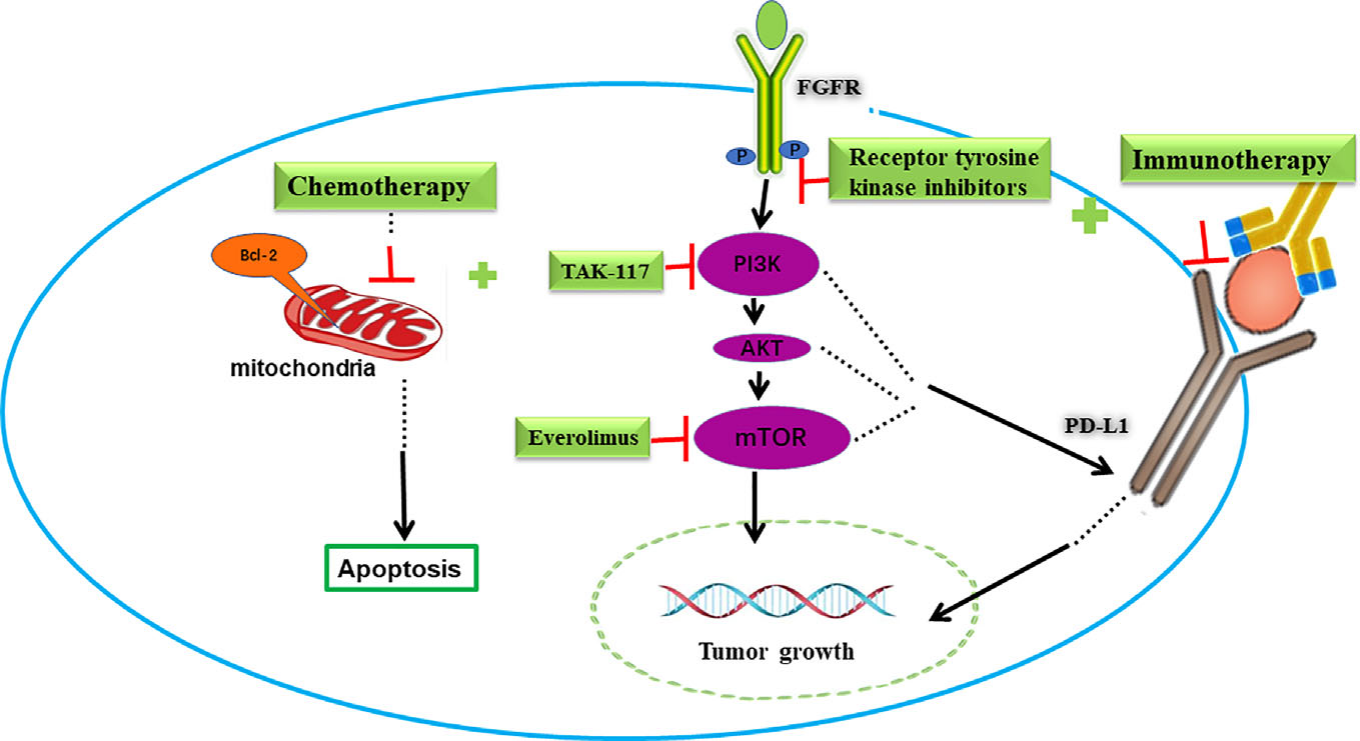
Combination of targeted therapy with immunotherapy or chemotherapy in bladder cancer. FGFR and PI3K/AKT/mTOR signing pathways are potential targets in bladder cancer. Blocking FGFR or PI3K/AKT/mTOR pathway decreased PD-L1 levels and increased immunotherapy response. On the other hand, these targeted drugs increased the pro-apoptotic effect and cytotoxic effect of chemotherapy drugs.

Targeted therapies have not been added to the crucial backbone of the treatment in bladder cancer so far. Comprehensive analyses of MIBC samples, expanding from 131 to 412, identified significantly mutated genes, including FGFR3, phosphatidylinositol 3 kinase (PI3K)/protein kinase B (AKT) pathway, Peroxisome proliferator-activated receptors (PPAR) γ mutations, DNA repair, p53 and cell cycle (7, 62). The good news is that, in April of 2019, the US FDA approved erdafitinib as an oral pan-FGFR-targeted agent indicated for metastatic urothelial cancer (UC) patients with susceptible FGFR3 or FGFR2 alterations (12). Despite genomic instability, molecular heterogeneity, and pathway redundancy still presenting challenges to targeted therapies in bladder cancer, researchers are making strategies to improve efficacy. Here, we present the combination effects of targeted therapies with other drugs in preclinical settings (**Figure 2**).

### Combination of FGFR Inhibitors With Immunotherapy

Erdafitinib, as the first TKI approved in UC therapy, has been demonstrated to be beneficial in clinical trials. Similar to other targeted drugs, toxicity and drug responses become concerns. Research has suggested that the presence of an antitumor T-cell response is fundamental for the activity of immunotherapy (63). Recently, Sweis et al. showed that UC can be divided into T-cell-inflamed and non-T-cell-inflamed subtypes. The latter phenotype correlated with a resistance to ICIs, but was also linked to FGFR3 mutation, providing a rationale for combining FGFR inhibitors and anti-PD-1/PD-L1 (64). Preliminary data in the FIERCE-22 study showed that the ORR was 36% in the overall population, and a response was observed in both wild type (ORR33%) and mutated (ORR 43%) FGFR3 patients receiving vofatamab (FGFR3 inhibitor) and pembrolizumab (anti-PD-1) (65). Powles et al. conducted a phase I study (NCT02546661) enrolled with platinum-resistant and ICI naïve patients with A/M UC harboring FGFR mutations (66). However, the results showed that AZD4547 (FGFR1-3 inhibitor) plus durvalumab increased response modestly compared to AZD4547 alone (n=21, ORR 29% versus n=15, ORR 20%, respectively), suggesting that the tumor mutations burden might contribute rather small differences to ICI response. A phase Ib/II study of rogaratinib combined with atezolizumab in patients with untreated FGFR-positive UC is currently in progress (NCT03473756). Likewise, the safety and efficacy of erdafitinib plus JNJ-63723283 (an anti-PD-1 monoclonal antibody) are investigated by a phase Ib/II study (NCT03473743) in advanced UC patients with FGFR gene alterations.

FGFR inhibitors may induce tumor environment changes and sensitize ICIs. However, FGFR alterations in UC contribute to intrinsic resistance to FGFR inhibitors. Thus, some patients with FGFR point mutations or fusions did not respond to erdafitinib or other FGFR inhibitors. Lima et al. observed activating FGFR3 mutants and FGFR3-TACC3 (transforming acidic coiled-coil containing protein 3) fusion constitutively elevated Src levels (67). Low dose dasatinib sensitized UC to FGFR TKIs, implying that the combination of FGFR with Src inhibitors may overcome intrinsic resistance compared with FGFR TKI monotherapy.

### Combination of PI3K/AKT/mTOR Inhibitors With Immunotherapy or Chemotherapy

The PI3K/AKT/mammalian target of rapamycin (mTOR) pathway is an important signal pathway closely related to protein synthesis, cell growth, survival and tumorigenesis (68). The deregulation of this signaling pathway is present in 42% of UC, including mutations, copy number alterations, or RNA expression changes (62). Despite the frequent deregulation, clinical trials using PI3K/mTOR inhibitors have not shown prominent success. The PIK3CA gene is an oncogene that implicates the overactivation of the PI3K/AKT/mTOR pathway. Recurrent somatic mutations of PIK3CA increase the activity of PI3Ks and the loss of phosphatase and tension homolog (PTEN, a tumor suppressor that inhibits PI3K) also can result in the overactivation of the PI3K/AKT/mTOR pathway (69).

A study in human glioma suggested that the loss of PTEN and the consequent upregulation of the PI3K-AKT pathway increased the expression of PD-L1 post-transcriptionally, thus promoting immune resistance (70). Additionally, other reports validated this resistance in melanoma, prostate and breast cancers, making the inhibition of PI3K-AKT pathway a potential strategy to overcome immunotherapy-resistance (71, 72).

Recently, a study showed that the PIK3CA mutation correlated with immune cell infiltration. In human urothelial bladder cancer samples, the expression of the immune gene signature which represents the immune cell infiltration in PIK3CA-mutated tumors was significantly lower than that of wild type counterparts. It means PIK3CA-mutated tumors may show lower response to ICIs therapy. In a humanized mouse model of bladder cancer with PIK3CA mutation, the treatment of BKM120(a pan-PI3K inhibitor) increases the expression of chemokines and immune genes. Notably, compared to the single treatment, BKM120 combined with Nivolumab (an anti-PD-1antibody) significantly inhibited the growth of PIK3CA-mutated tumors (73). And a clinical trial is now investigating the therapeutic promise of durvalumab (an antibody that blocks PD-L1) in combination with vistusertib (AZD2014) in MIBC patients with rapamycin-insensitive companion of mTOR (RICTOR) amplification, or tuberous sclerosis complex (TSC)1/1 mutation. (NCT02546661 module E)

Chemotherapy drugs kill tumor cells primarily through the induction of apoptosis. The activation of PI3K/AKT/mTOR pathway in tumor cells reduces the pro-apoptotic effect and the cytotoxic effect of chemotherapy drugs, leading to resistance (74). Therefore, inhibition of this signaling pathway may enhance the sensitivity of chemotherapy drugs.

Zeng et al. reported that in the patient-derived xenograft models with a PI3K mutation or amplification, the combination groups (pictilisib with cisplatin and/or gemcitabine) achieved significant delay of tumor growth and increased survival compared with any single drug (pictilisib/cisplatin/gemcitabine) (75). When combining TAK-228 (an oral mTORC1/2 inhibitor) with TAK-117 (PI3Kα inhibitor) or with paclitaxel, strong synergistic effect was also observed in preclinical bladder cancer models (76). These results facilitate a clinical trial to investigate efficacy of TAK-228 plus paclitaxel in patients with metastatic bladder cancer (NCT03745911). Similar results were obtained through combining the PI3K/mTOR dual inhibitor NVP-BEZ235 with cisplatin in osteosarcoma, triple negative breast cancer and bladder cancer (77, 78). Moon et al. demonstrated that when NVP-BEZ235 was used in combination to treat cisplatin-resistant T24R2 cells, the IC_50_ of cisplatin and NVP-BEZ235 could be reduced by 3.6- and 5.6-fold, respectively (79).

However, the results of clinical trials seem to be inconsistent. A phase II trial of BEZ235 evaluated in 20 advanced bladder cancer patients after failure of platinum-based therapy conveyed a modest activity but a hostile toxicity with 50% grade 3-4 adverse effects (80). Single-agent mTOR inhibitor temsirolimus and everolimus also showed limited efficacy (81, 82), whereas one patient carrying a TSC 1-inactivating mutation treated with everolimus had notable tumor shrinkage and durable response, suggesting the blockade of the PI3K/mTOR axis could improve outcome in some specific patients (83). The role of paclitaxel/everolimus combination in metastatic UC was investigated in the phase II AUO AB 35/09 trial, and the results were modest (PFS was 2.9 months, 3 months and ORR 13%) (84). Thus, better understanding of the molecular landscape of these tumors and more precise patient selection might be helpful for a more rational design of combination therapy.

## Chemotherapy

Chemotherapy is a routine treatment in cancer. There are two different chemotherapeutic routes in bladder cancer, including intravesical BCG/MMC for NMIBC and systemic chemotherapies for MIBC. Although it has brought benefits to patients in the past decades, intolerant toxicity needs to be improved. Novel combinations of chemotherapeutic drugs with others are studied.

### Combination of Interleukin-15 Super-Agonist With BCG

Interleukin-15 (IL-15) is implicated in the development, proliferation, and activation of effector NK cells and CD8+ memory T cells. However, its short half-life, high dose requirement for clinical activity, and prohibitive toxicity represent barriers for successful clinical trial development (85). To overcome these shortcomings, ALT-803 was developed as a novel fusion complex. Recombinant IL-15, a super-agonist due to an activating N72D mutation, is bound to the soluble receptor IL-15RαSushi-Fc. This complex has improved bioavailability, increased serum half-life, longer retention in lymphoid organs, and *in vivo* biological activity up to 25 times that of native IL-15. ALT-803 has demonstrated potent immunostimulatory effects in terms of triggering a local cytokine response as well as activating NK and CD8+ T cells in animal models (85). In a carcinogen-induced rodent NMIBC tumor model, intravesical ALT-803 plus BCG treatment reduced tumor burden by 46% vs ALT-803 (35%) or BCG (15%) alone (86). An ongoing multicenter, open-label, single-arm phase II trial (QUILT-3.032) is evaluating ALT-803 in combination with BCG administered *via* intravesical instillation in patients with BCG-unresponsive NMIBC (NCT03022825). Recently presented preliminary results indicate that six of the seven evaluable patients with BCG-unresponsive carcinoma *in situ* achieved a CR at the 12-week response assessment (87).

### Combination of Chemotherapeutic Drug MMC With BCG/Gemcitabine

BCG and Mitomycin C (MMC) are representatives of clinical intravesical immunotherapy and chemotherapy drugs for NMIBC.

A randomized prospective trial involved 407 patients with intermediate- to high-risk NMIBC found that sequential combination of MMC plus BCG is more effective but more toxic than BCG alone. Thus, it was recommended to patients with a high likelihood of recurrence, such as those with recurrent T1 tumors (88 **)**. Another study including 151 patients with high-risk NMIBC demonstrated outstanding efficacy for sequential BCG and EMDA-MMC (Electro Motive drug administration of MMC). The complete-response rate was 87%, with 86% and 93% remaining disease-free at one and two years respectively which is better than any previously published outcomes in this disease (89).

Gemcitabine is a pyrimidine analogue that incorporates into actively replicating DNA and thereby prevents further synthesis, whereas MMC cross-links DNA moieties to prevent synthesis (90). In addition, MMC is a vesicant to the urothelium, which could increase permeability to subsequent gemcitabine administration through its irritating action. So, it is available to combine MMC with gemcitabine as a possibly effective way to enhance mutual absorption and control tumor progression (91). Sequential intravesical gemcitabine and MMC in NMIBC patients appeared to be well tolerated and yielded a response in a good proportion of patients with recurrent BCG refractory bladder cancer or who are not surgical candidates (92 **)**.

Furthermore, combination of MMC with other novel methods also suggests improved treatment effect. Proliferating cell nuclear antigen (PCNA) is an essential scaffold protein in multiple cellular processes including DNA replication and repair (93 **)**. More than 200 proteins, many involved in stress responses, interact with PCNA through the AlkB homologue 2 PCNA-interacting motif (APIM), including several proteins directly or indirectly involved in repair of DNA interstrand crosslinks (ICLs) (94 **)**. Gederaas et al. targeted PCNA with a novel peptide drug containing the APIM sequence, ATX-101, to inhibit repair of the DNA damage introduced by the chemotherapeutics. Results showed that ATX-101 increased the anticancer efficacy of the ICL-inducing drug MMC and ATX-101 given intravesically in combination with MMC penetrating the bladder wall and further reducing the tumor growth in both the slow growing endogenously induced and the rapidly growing transplanted tumors (95 **)**. Survivin inhibits apoptosis and enables tumor cells to escape from therapy-induced senescence. High expression of survivin is associated with bladder cancer aggressiveness and recurrence. Cui et al. demonstrated that silencing survivin enhanced activity of MMC in human bladder RT4 xenografts, representing a potentially useful chemo-gene therapy for bladder cancer (96 **).** These data indicate that combination of MMC can be a useful approach to improve the effect of chemotherapy.

### Platinum-Based Combination Treatment

Chemotherapy with MVAC (methotrexate, vinblastine, doxorubicin, and cisplatin) or GC (Gemcitabine plus cisplatin) are considered the gold standard of care for MIBC. To improve efficacy and reduce toxicity, clinical researchers are still trying to develop new combinations. Taxanes, including paclitaxel, docetaxel and derivatives with taxane structure, are well-known antitumor drugs. Combination of platinum with taxanes has emerged as an alternative option for MIBC patients (97).

Apart from combination of clinically available chemotherapeutic agents, several preclinical trials focusing on novel mechanisms that can improve efficacy and sensitize chemoresistance of cisplatin have been studied. Obatoclax, a BH3 mimetic which inhibits pro-survival Bcl-2 family members, can inhibit cell proliferation, promote apoptosis, and significantly enhance the effectiveness of cisplatin in MIBC cells *via* inhibiting Bcl-2 and Bcl-xL and decreasing cyclin D1 and Cdk4/6 expression levels (98 **)**. This finding can help validate Obatoclax as a cell cycle inhibitor and increase the attractiveness of Obatoclax as an anti-cancer drug. Enzalutamide, a synthetic androgen receptor (AR) signaling inhibitor, synergistically inhibited growth of bladder cancer cells more efficiently when combined with cisplatin. This supports the feasibility for future investigation of AR antagonists in combination with standard chemotherapy in MIBC (99 **)**. Besides, obtained data *via* an epigenomic approach suggested that Homeobox A9 promoter methylation could serve as a potential predictive biomarker and decitabine might sensitize resistant tumors in patients receiving cisplatin-based chemotherapy, but clinical trials are needed to confirm this conclusion (100 **)**.

## Discussion

As we stated above, due to the efforts of the scientific community, the management of bladder cancer, especially for advanced patients, has made great progress recently despite the slow rate of development (101). Two milestones, the application of ICIs and approval of oral FGFR-TKI erdafitinib have made tremendous progress (12, 17). ICIs bring a revolutionary impact on patients with durable outcomes in a subset of individuals with tolerable adverse event profiles (12). More importantly, marked advances to understand the molecular interplay within the immune environment have been generated in the past decades (63, 64). Thus, combination of immunotherapy with other therapies has been designed to improve efficacy, increase responses and reduce toxicity. Simultaneously, antibody-drug conjugates represent a new therapeutic modality in urothelial cancer. Enfortumab vedotin (EV), is the first antibody-drug conjugate, which gained approval in December, 2019 in advanced UC. Clinical trials seek to improve its efficacy *via* novel combinations such as combining EV with immunotherapy drugs. There are also new ADCs under investigation and showing promise. Although the results show that combination therapies produce encouraging outcomes, there are still several unsolved issues. First, the detailed mechanisms of each ICIs need to be investigated. Second, biomarkers are required to analyze through molecular diagnosis helping in understanding patient-specific immune-suppression. Third, toxicity should be tolerable with proper drug doses and irradiation duration time.

The approval of FGFR-TKI erdafitinib made a breakthrough for metastatic bladder cancer targeted therapy. Combination of FGFR-TKI with ICIs has the potential to overcome drug-resistance barriers as well as augment immunogenicity of the tumor – even in patients who lack response to ICIs monotherapy (65). As pointed out in the previous section, PI3K/AKT/mTOR pathway plays an important role in bladder tumorigenesis, conferring PI3K/AKT/mTOR potential targets in bladder cancer. Unfortunately, clinical results of these targeted inhibitors, alone or in combination, are not very encouraging so far (80–82). The possible reason is that the molecular landscape and pathophysiology of patients were not fully and deeply understood. Thus, assays such as genome sequencing and immunohistochemical analyses could be employed to select appropriate patients.

Intravesical drugs including BCG and MMC, the clinical guidelines recommended for NMIBC after tumor resection have been clinically used for a long time (102, 103). The past decades witnessed their benefits to patients. However, recurrence has been a big challenge all the time for these administration strategies. New multiagent intravesical chemotherapy regimens for instance, either interleukin-15 super-agonist or MMC with BCG have been developed in recent years, dramatically enhancing antitumor activity of BCG (2). Either GC or MVAC is well-accepted neoadjuvant chemotherapies for MIBC (104). As stated above, pathologic information of patients largely helps medical doctors to make the decision to choose either GC or MVAC to treat bladder cancer patients, depending on molecular characteristics of individuals (105).

In addition, intravesical administration route is a particular way for bladder cancer treatment due to the unique physiological features of urinary bladders. The strategy increases the local concentrations within the bladder and avoids the systemic toxicity of drugs. Due to the possible interactions among drugs, the physical and chemical profiles of drugs should be carefully considered when combining.

## Conclusions

**Figure 3:**
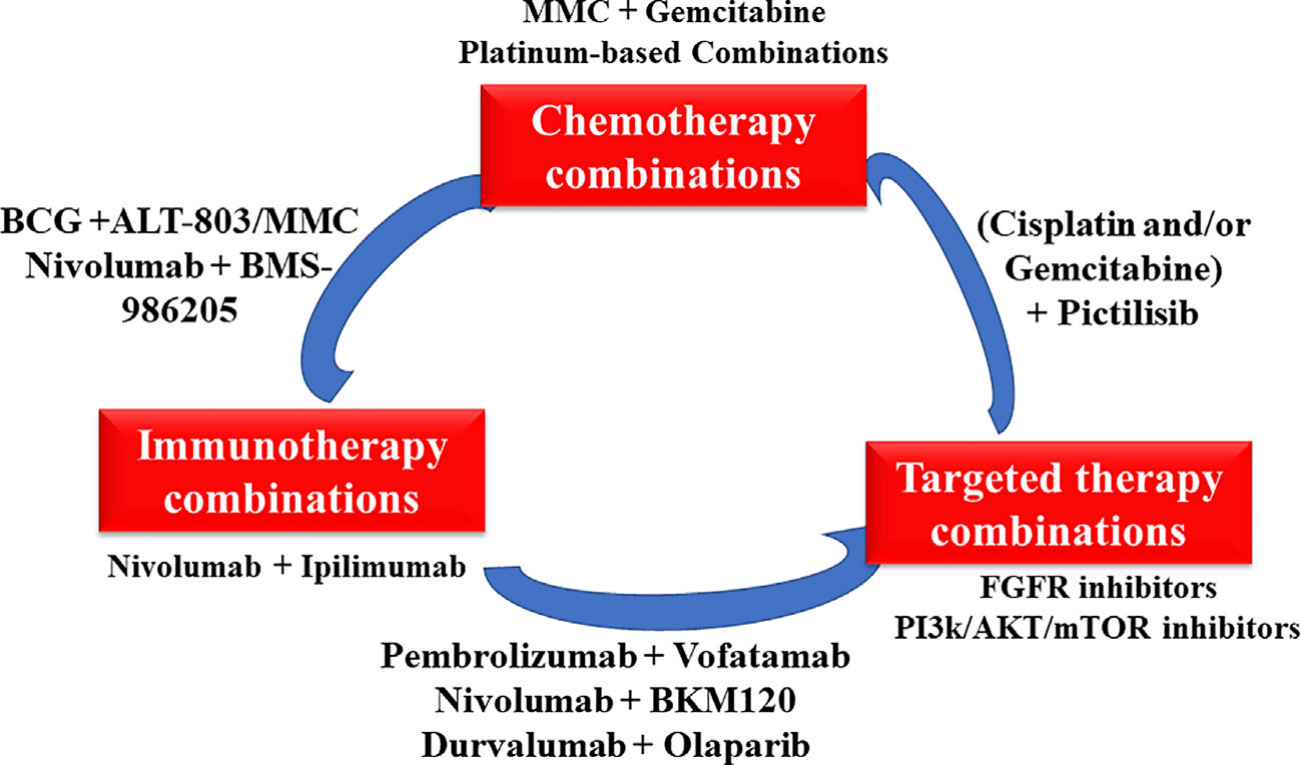
The overview of various novel combinations in bladder cancer.

In conclusion, combination therapy is a classic and proven strategy to improve patients’ survival. Many combination therapies as shown in **Figure 3** such as dual immunotherapies and alternate ICIs with targeted therapies are understudied, holding considerable promise for treating bladder cancer. The revolution of bladder cancer treatment will keep moving forward with a good understanding the biology of bladder cancer based on rapid drug development.

## Author Contributions

MP and XiY designed this study, wrote and revised the manuscript. DX, YB, JL, XuY, and SL prepared the literatures and drafted the manuscript. All authors contributed to the article and approved the submitted version.

## Funding

This research was funded by National Natural Science Foundation of China (81703008,81874212), Huxiang High-Level Talent Innovation Team (2018RS3072), Major Scientific and Technological Projects for Collaborative Prevention and Control of Birth Defect in Hunan Province (2019SK1012).

## Conflict of Interest

The authors declare that the research was conducted in the absence of any commercial or financial relationships that could be construed as a potential conflict of interest.

## Abbreviations

ADCs, antibody-drug conjugates; AKT, protein kinase B; APIM, AlkB homologue 2 PCNA-interacting motif; AR, androgen receptor; BCG, bacillus Calmette‑Guerin; CTLA-4, cytotoxic T lymphocyte-associated antigen-4; DC, docetaxel with cisplatin; DDR, DNA damage repair; DNMT1, DNA methyltransferase1; EV, enfortumab vedotin; FDA, food and drug administration; FGFR-TKI, fibroblast growth factor receptor tyrosine receptor inhibitor; GC, Gemcitabine plus cisplatin; ICIs, immune checkpoint inhibitors; ICLs, interstrand crosslinks; IFN γ, interferon gamma; IL-15, interleukin-15; IDO, Indoleamine 2,3-dioxygenase; MIBC, muscle invasive bladder cancer; MMC, Mitomycin C; MVAC, methotrexate, vinblastine, doxorubicin and cisplatin; mTOR, mammalian target of rapamycin; NMIBC, non-muscle invasive bladder cancer; NK, natural killer; ORR, objective response rate; OS, overall survival; PARP, poly ADP-ribose polymerase; PD-1, programmed death-1; PD-L1, programmed death-1 ligand 1; PCNA, proliferating cell nuclear antigen; PFS, progression-free survival; PI3K, phosphatidylinositol 3 kinase; PLUMMB, pembrolizumab in muscle-invasive/metastatic bladder cancer; polyICLC, polyinosinic-polycytidylic acid-poly-l-lysine carboxymethylcellulose; PPAR, peroxisome proliferator-activated receptors; pT0, the pathological T0 rate; PTEN, gene of phosphate and tension homology deleted on chromosome ten; RICTOR, Rapamycin-insensitive companion of mTOR; RT, radiation therapy; SoC, standard of care; TAAs, tumor-associated antigens; TACC3, transforming acidic coiled-coil containing protein 3; TAMs, tumor-associated macrophages; TCC, transitional cell carcinoma; Tregs, regulatory T cells; TIL, tumor-infiltrating lymphocytes; TSC, tuberous sclerosis complex; UC, urothelial carcinoma.
